# *Arundo donax* L Processing in Catalyzed Butanol–Water Media in the Scope of Lignocellulose Biorefineries

**DOI:** 10.3390/polym15061553

**Published:** 2023-03-21

**Authors:** Sandra Rivas, Rebecca Baldassari, Juan Carlos Parajó, Anna M. Raspolli Galletti

**Affiliations:** 1 Faculty of Science, Chemical Engineering Department, University of Vigo (Campus Ourense), Polytechnical Building, As Lagoas, 32004 Ourense, Spain; 2 CINBIO, University of Vigo (Campus Lagoas-Marcosende), 36310 Vigo, Spain; 3 Department of Chemistry and Industrial Chemistry, University of Pisa, Via G. Moruzzi, 13, 56124 Pisa, Italy

**Keywords:** *Arundo donax*, fractionation, butanol–water, cellulose, hemicelluloses

## Abstract

*Arundo donax* L samples, before or after aqueous extraction to remove extractives, were subjected to chemical fractionation in H_2_SO_4_-catalyzed mixtures of 1-butanol and water. The partial miscibility of 1-butanol and water at room temperature allowed the separation of the three major feedstock components in separate streams (lignin, accumulated in the 1-butanol-rich phase; hemicellulose-derived products, accumulated in the aqueous acidic phase; and cellulose, present in the solid phase). The effects of selected variables (temperature, catalyst concentration, reaction time and 1-butanol content of the reaction media) on variables measuring the solid recovery yield and the compositions of phases from fractionation were measured. Using water-extracted *A. donax* L as a substrate, the best operational conditions enabled 93.2% hemicellulose removal and 85.4% delignification with limited cellulose solubilization (15%). The experimental results provided key information to assess the proposed process in the scope of biorefineries.

## 1. Introduction

Lignocellulosic materials (LCMs) are a renewable resource that is expected to play a key role in the development of a sustainable bioeconomy. There are diverse types of LCM, from raw vegetal biomass to industrial by-products or wastes. The LCM major components are of polymeric nature, including polysaccharides (cellulose and hemicellulose) and an aromatic polymer (lignin).

*Arundo donax* L (AD), also named giant cane or giant reed, is a non-edible, herbaceous plant present in many geographical areas of the world. AD presents a number of advantages as an industrial feedstock, including high biomass productivity, flexibility to grow under a variety of climatic conditions with little or no irrigation, low fertilization requirements and suitability for cultivation in set-aside lands not suitable for food crops [1,2,3,4,5].

LCMs present a complex structure, in which hemicelluloses and lignin form a tri-dimensional matrix surrounding the cellulose fibers. Since these constituents have different properties, LCMs can be conveniently utilized on the basis of the lignocellulose biorefinery approach, based on the selective separation (“fractionation”) of the major components. Upon fractionation, the polymeric LCM constituents (or fractions derived from them) appear in separate streams, allowing their individual benefit. Using this idea, AD has been employed as a raw material for the industrial manufacture of cellulose pulp, paper, added value bioproducts and biofuels [6,7,8].

The biorefinery approach may be implemented in multiple ways, including the ones based on the utilization of organosolvents (“organosolv fractionation”). A number of organic solvents have been employed in this field, including short chain acids, alcohols, aromatics, ethers, ketones and lactones [9].

Although most organosolv studies employed reaction media made up of aqueous ethanol, the utilization of 1-butanol–water mixtures shows an interesting feature: At room temperature, 1-butanol and water are partially miscible, but they become totally miscible at typical reaction temperatures [10]. As a consequence, the reactions take place in a single liquid phase, which separates in organic and aqueous layers when the reaction medium is cooled after fractionation.

The fractionation effects achieved in aqueous 1-butanol can be summarized as follows [11,12]: Cellulose remains in solid phase; the soluble lignin-derived compounds are accumulated in the organic phase; and hemicelluloses (or their derived products) may appear mainly either in solid phase (when operation is performed under mild conditions) or in liquid phase (as reaction products such as oligosaccharides, monosaccharides and/or monosaccharide-decomposition products, when operation is conducted under harsh conditions). This means that the separation of the three polymeric lignocellulose constituents can be achieved in a single treatment (“one-pot” approach), defining an efficient framework for the sustainable manufacture of bio-based chemicals.

Adding catalysts (typically, mineral acids at low concentrations) to aqueous 1-butanol increases the degree of feedstock solubilization achieved under defined conditions, as well as the yields of monosaccharides and/or monosaccharide-decomposition products (furans). The product distribution depends on the operational conditions, which have to be tuned carefully for achieving the desired effects. Catalyzed mixtures of 1-butanol and water have been used for fractionating diverse LCMs, as discussed in Section 3.2.

Considered as a fractionation agent, 1-butanol presents additional advantages, such as green character (based on the evaluation of the environmental risks), obtainable from LCM by bacterial fermentation of polysaccharide-derived sugars or by Guerbet condensation of bioethanol [13], high partition coefficients respect to important biomass-derived chemicals (i.e., furans), favorable relative volatility respect to a number of biorefinery products, good ability for lignin solubilization with formation of sulphur-free lignin fragments suitable for further chemical conversion into aromatic chemicals, and recovery and recycling facilitated by the biphasic nature of the reaction media at room temperature [12,14,15,16].

The organosolv fractionation of LCM has been carried out in diverse types of reactors. Among them, the ones heated with microwaves stand out for their ability to allow an improved operation [17], particularly when fast heating profiles boost the target product yields. Moreover, microwave reactors are energy effective, easy to operate and limit the temperature gradients inside the media.

This study deals with the fractionation of AD (before or after removal of water-soluble extractives) in acid-catalyzed mixtures of 1-butanol and water, operating in a microwave-heated reactor. The effects caused by treatments performed under diverse operational conditions were assessed in terms of the compositions of the phases from treatments, the degrees of substrate solubilization and additional variables measuring the separation selectivity.

## 2. Materials and Methods

### 2.1. Raw Material

AD was kindly provided by the Institute of Life Sciences (Scuola Superiore Sant’Anna, Pisa, Italy). AD was collected from long-term field trials at the Interdepartmental Center for Agro-Environmental Research (University of Pisa), located in San Piero a Grado (latitude 43°68′ N, longitude 10°35′ E). Before use, AD was kept at 60 °C in a ventilated furnace for the time needed to achieve a constant weight.

### 2.2. Removal of Water-Soluble Extractives

AD was mixed with water (10 kg water/kg dry AD) in a 600 mL stainless steel, stirred reactor (from Parr Instrument Co., Moline, IL, USA), heated up to reach 130 °C and cooled immediately. The solid free from aqueous extractives (EF.AD) was separated from the liquid phase by vacuum filtration and employed for solid yield determination and compositional analysis (see below).

### 2.3. Organosolv Fractionation

Samples of AD or EF.AD were subjected to organosolv processing (OP) in media made up of 1-butanol, water and sulfuric acid (acting as a catalyst), using a Microwave Accelerated Reaction System (MARS 6, CEM Corporation, Matthews, NC, USA). Treatments were performed at a fixed liquid to solid ratio (LSR) of 15 mL/g of oven-dry solid substrate (AD or EF.AD) under diverse operational conditions (see below), yielding solids denoted OP.AD (when AD was the substrate) or OP.EF.AD (when EF.AD was the feed material). OP.AD and OP.EF.AD were separated from the fractionation media by filtration, washed (first with 1-butanol and then with water), employed for solid yield determination and analyzed using the methodology detailed below. The aqueous and organic phases from organosolv treatments were separated using a separation funnel and analyzed individually (see below). The definitions of the variables employed in the discussion are as follows:

(1) $$Solid\;yield\;\left(SY,\;\%\right)=100\times \frac{Mass\;of\;dry\;solid\;after\;treatment_{}}{Mass\;of\;dry\;solid\;before\;treatment}$$

(2) $$\begin{array}{l} \%\;Glucosyl\;group\;solubilization\;\left(\%GnSol\right)\\ =100\times \frac{Mass\;of\;glucosyl\;groups\;in\;the\;solid\;subjected\;to\;treatment-Mass\;of\;glucosyl\;groups\;in\;the\;treated\;solid}{Mass\;of\;glucosyl\;groups\;in\;the\;solid\;subjected\;to\;treatment} \end{array}$$

(3) $$\begin{array}{l} \%\;Hemicellulose\;solubilization\;\left(\%HSol\right)\\ =100\times \frac{Mass\;of\;hemicelluloses\;in\;the\;solid\;subjected\;to\;treatment-Mass\;of\;hemicelluloses\;in\;the\;treated\;solid}{Mass\;of\;hemicelluloses\;in\;the\;solid\;subjected\;to\;treatment} \end{array}$$

(4) $$\begin{array}{l} \%\;Lignin\;solubization\;\left(\%LSol\right)\\ =100\times \frac{Mass\;of\;lignin\;in\;the\;solid\;subjected\;to\;treatment-Mass\;of\;lignin\;in\;the\;treated\;solid}{Mass\;of\;lignin\;in\;the\;solid\;subjected\;to\;treatment} \end{array}$$

### 2.4. Analytical Methods

AD, EF.AD, OP.AD and OP.EF.AD samples were assayed for extractives using the NREL/TP-510-42619 method [18] and for structural polysaccharides, acetyl substituents, Klason lignin (denoted KL) and acid soluble lignin (denoted ASL) using the NREL/TP-510-42618 method [19]. The total lignin content was calculated as the sum of the contributions of KL and ASL. The liquid phases from the NREL/TP-510-42618 assays, containing saccharides, acetic acid and sugar-degradation products (furans) were assayed by HPLC, using an Agilent 1200 Series instrument (Agilent Technologies, Inc., Santa Clara, CA, USA) equipped with a refractive index detector (RID) and a diode array detector (DAD) and fitted with a 300 × 7.8 mm BioRad Aminex HPX-87H column, kept at 50 °C and eluted with 0.6 mL/min of 0.003N H_2_SO_4._ All analyses were made in triplicate. The liquid phases from the diverse reaction media were analyzed using the same HPLC method. Additionally, the liquid samples from OP processing were subjected to a quantitative posthydrolysis (with 4% H_2_SO_4_ at 121 °C for 20 min) before HPLC analysis to allow the quantitation of saccharides and acetic acid (based on the amounts of monosaccharides and acetic acid generated upon posthydrolysis). After dilution and centrifugation, aliquots of organic phases were analyzed for monosaccharides, acetic acid and furans using the same HPLC method.

## 3. Results and Discussion

### 3.1. AD Composition and Aqueous Extraction

**Figure 1:**
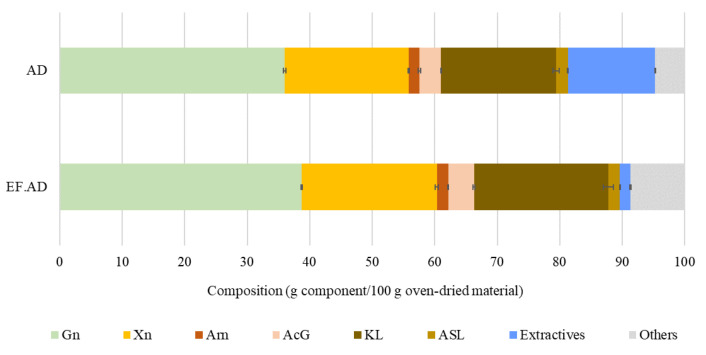
Average composition of AD and water-extracted AD (EF.AD), expressed as g of component per 100 g oven-dried material ± standard deviation. Nomenclature: anhydroglucose units, G_n_; anhydroxylose units, X_n_; anhydroarabinose units, Ar_n_; acetyl groups, AcG; Klason Lignin, KL; Acid Soluble Lignin, ASL.

The average composition of the AD lot employed in experiments, expressed as g of component per 100 g of dry biomass ± standard deviation, is shown in Figure 1. AD was mainly made up of anhydroglucose units (considered as cellulose and denoted G_n_), which accounted for 35.99% of the raw material. The AD hemicellulose content was 25.05% and corresponded to anhydroxylose units (denoted X_n_), anhydroarabinose units (denoted Ar_n_) and acetyl groups (denoted AcG). The total lignin, calculated as the sum of KL and ASL, accounted for 20.3%; whereas extractives (13.95%) and other (4.74%, calculated by difference) were also present. These results are within the ranges of compositional data summarized by Corno et al. [1] (cellulose, 29.2–39.6; hemicelluloses, 14.5–32.0; lignin, 19.2-24.3 wt%). The results indicated that the major AD hemicellulose polymer was heteroxylan, made up of anhydroxylose units substituted with acetyl groups and arabinosyl units. It can be noted that AD may contain anhydrosugars different from anhydroglucose, anhydroxylose and anhydroarabinose, which have not been considered in this study owing to their small proportions. For example, Scordia et al. [20] reported 0.66% galactan, 0.12% mannan and 0.06% rhamnan for AD samples cultured in Italy; whereas Shatalov and Pereira [21] reported contents below 1% for both mannose and glucose from the quantitative acid hydrolysis of AD grown in Greece.

AD was treated with water at 130 °C to yield EF.AD, the second solid substrate employed in this study. The solid yield, expressed in oven-dry basis, was 88.7 g EF.AD/100 g AD. Figure 1 shows the EF.AD composition, expressed as g of component per 100 g of oven-dry EF.AD ± standard deviation. As expected, the mild extraction conditions did not result in significant solubilization of the structural components (cellulose, heteroxylan and Klason lignin), but the non-polar extractives retained in EF.AD accounted for just 10.5% of the total extractives in AD. Since the extractive fraction may interfere in the fractionation stage (for example, by yielding condensation and/or soluble products that decrease the potential of the reaction products for further processing), and taking into account the structural alteration caused by the aqueous extraction (i.e., swelling), it can be concluded that EF.AD should be a more favorable fractionation substrate than AD. To assess this point, both AD and EF.AD were employed as substrates for organosolv processing (see below).

The liquid phase from the aqueous extraction presented polysaccharide-derived compounds at little concentrations, including monosaccharides (1.41 g glucose/L, 0.2 g xylose/L, 0.07 g arabinose/L), acetic acid (0.09 g /L), and higher soluble saccharides, denoted HSS. This latter fraction included 0.06 g anhydroglucose units/L, 0.15 g anhydroxylose units/L, 0.11 g anhydroarabinose units /L and 0.040 g of attached acetyl groups/L.

### 3.2. Organosolv Treatments

#### 3.2.1. AD Processing

The utilization of biphasic systems represents an efficient and potentially profitable alternative for LCM processing, based on their ability to exploit the differences in polarity of the diverse reaction products [22].

1-butanol has been considered as a green solvent, based on the comparative evaluation of both environmental and health hazards [23]. This information is also coherent one included the Pfizer, GSK, and Sanofi solvent selection guides [24]. On the other hand, the fact that 1-butanol can be produced from LCM increases its interest as a solvent for biorefineries.

Organosolv fractionation can be carried out in the absence or in the presence of catalysts. A number of heterogeneous and homogeneous catalysts have been used in literature. Among the homogeneous catalysts, the most employed ones have been Brönsted acids, because they release hydronium ions upon dissociation, which are the active catalytic species allowing the breakdown of ether bonds making part of polysaccharides and lignin. Strong mineral acids (such as HCl or H_2_SO_4_) are the most employed catalysts for LCM deconstruction in organosolv media. In this work, H_2_SO_4_ was preferred to HCl owing to its less corrosive character.

Both raw and pre-processed LCMs have been considered as substrates for treatments with 1-butanol–water mixtures. The raw LCMs tested include softwoods and hardwoods [12,25,26,27], herbs [12,27], walnut shells [25,27,28], straws [29,30,31], rice husks [28,30,32], sorghum culms [33,34], banana stalks and jute sticks [30].

In these studies, the operational conditions (temperature, reaction time, solid charge, type of catalyst and relative amounts of catalyst–1-butanol–water) varied within wide ranges, depending on the desired effects (selective separation of the structural polymers, manufacture of cellulose fractions suitable as pulping products or as substrates for enzymatic hydrolysis, manufacture of pure lignins, production of valuable compounds from hemicelluloses, etc.). Regardless of this, some general conclusions can be drawn from these studies:

- Selective delignification leading to pure lignins can be achieved at the normal boiling point of 1-butanol or slightly higher temperatures in media containing high butanol proportions (up to 95%), operating with limited catalyst charges for prolonged reaction times. Under these conditions, lignin was extensively butoxylated, and butyl-xylosides are the major soluble products from hemicelluloses.
- Conditions of medium severity (defined by temperatures within the range 140–170 °C and/or intermediate catalyst concentrations and/or intermediate water proportions) resulted in higher polysaccharide solubilization. Soluble saccharides were the major hemicellulose-derived products, and increased cellulose dissolution is expected. The generation of soluble saccharides from cellulose and xylan involve the partial breakdown of the glycosidic bonds between anhydroglucose or anhydroxylose units, according to the following reactions:
  - (C_6_H_10_O_5_)_n_ + water → (C_6_H_10_O_5_)_m_ (m < n)
  - (C_5_H_8_O_4_)_p_ + water → (C_5_H_8_O_4_)_q_ (q < p)
- Harsher conditions may promote the production of monosaccharides (hexoses or pentoses) from oligosaccharides, as well as the monosaccharide dehydration into furans (5-hydroxymethylfurfural from hexoses, and furfural from pentoses), according to the following reactions:
  - (C_6_H_10_O_5_)_m_ + m H_2_O → m C_6_H_12_O_6_ → m C_6_H_6_O_3_ + 3m H_2_O
  - (C_5_H_8_O_4_)_p_ + p H_2_O → p C_5_H_10_O_5_ → p C_5_H_4_O_2_ + 3p H_2_O

**Figure 2:**
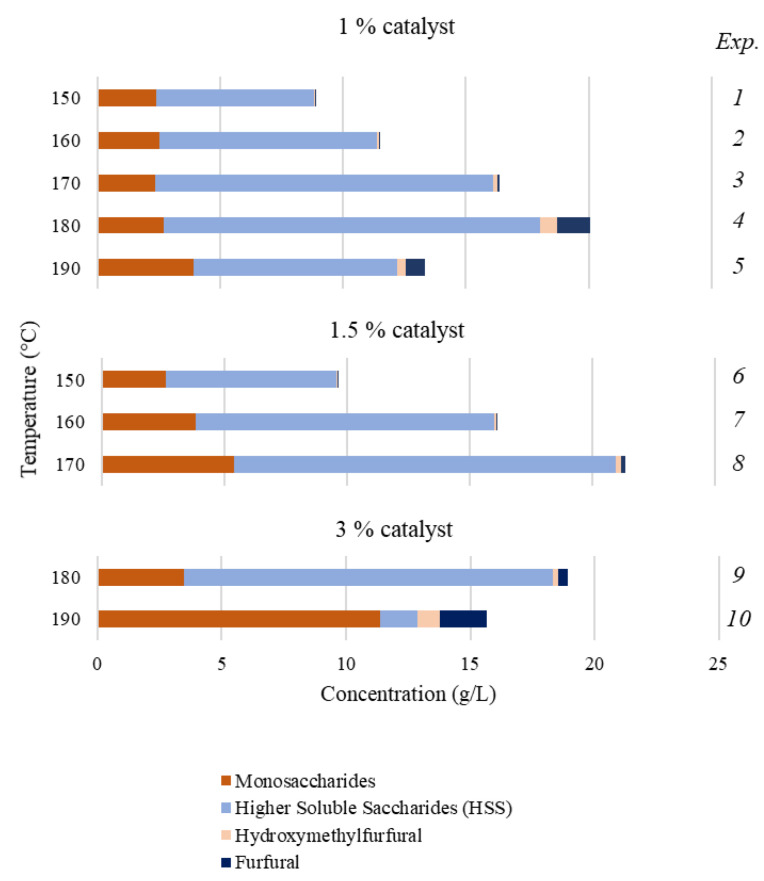
Concentrations of polysaccharide-derived products in the aqueous phases from AD processing obtained in experiments 1–10 in Table 1.

Based on both the above information and preliminary experimental results (data not shown), the experimental plan shown in Table 1 was defined to assess the effects of the H_2_SO_4_ concentration (1–3 wt%) and temperature (150–190 °C) on AD fractionation in media containing 1-butanol–water mixtures (33:67% *v*/*v*) operating with 15 mL liquid phase/g solid for 20 min. The same Table lists the experimental results achieved for the solid yields and compositions of the treated solids, whereas Table 2 presents the values calculated for the variables defined in Section 2.3. Additionally, the compositions of the aqueous phases from treatments (accumulating the polysaccharide-derived products) are presented in Figure 2.

The structure of the experimental plan was as follows: The first subset of assays (experiments 1 to 5) was performed to obtain a screening of the effects of temperature (within the range of operational interest) at the lowest catalyst concentration considered. The possible benefits resulting from operation under experimental conditions of intermediate severity were explored in a new subset of experiments (6 to 8) by using 50% more catalyst than in the previous trials, operating at 150–170 °C. Finally, harsh conditions were explored in experiments 9 and 10, in which the catalyst charge was doubled in respect to the one employed in experiments 6 to 8, and temperature was fixed at the highest values assayed (180 and 190 °C, respectively).

The test performed under the mildest conditions (experiment 1) resulted in a solid yield (75.5%) too high for practical purposes, coherent with a limited %LSol (17.1%), and poor %HSol (22.4%), indicating that better results could be obtained under harsher conditions. The major water-soluble reaction products were HSS and monosaccharides, all of them obtained at low concentrations (Figure 2).

Based on these findings, the severity was increased either by increasing temperature up to 160 °C while keeping the catalyst concentration constant (experiment 2 in Table 1) or by increasing the catalyst concentration up to 1.5% while keeping temperature at 150 °C (experiment 6 in Table 1). In comparison with experiment 1, the solids from both assays 2 and 6 showed improved G_n_ contents and higher percentages of lignin and hemicellulose removal, confirming an improved fractionation. In comparison with experiment 6, experiment 2 led to better results in terms of %LSol (32 in respect to 23.2%), hemicellulose removal (32.5% in respect to 28.6) and HSS concentration (see Figure 2). However, the solid yields (69.0 and 73.2% in experiments 2 and 6, respectively) and the limited degrees of both hemicellulose solubilization and delignification indicated that further severity increases could improve the results.

In further assays, higher temperatures were considered: 170, 180 and 190 °C in media with 1% catalyst (experiments 3, 4 and 5 in Table 1) and 160 and 170 °C in media with 1.5% catalyst (experiments 7 and 8 in Table 1). In these experiments, the highest substrate dissolution was achieved at the higher temperature assayed (experiment 5), leading to a processed solid (OP.AD), containing 77.7 G_n_, with 10.7% KL (corresponding to 78.6% lignin removal), extensive hemicellulose removal (%HSol, 92.8%) and 21.0 %G_n_Sol. In comparison, experiments 3, 4, 7 and 8 enabled 43.9–61.4% solid recovery, with OP.AD containing 49–66.6% G_n_ and 14.5–18.2% KL, keeping %LSol, %HSol and %G_n_Sol within the ranges 39.7–65.6, 50.9–79.1 and 14.9–18.7%, respectively. The conditions of experiment 8 (1.5% catalyst and 170 °C) were favorable for producing soluble hemicellulose-derived products, mainly in form of HSS, but also monosaccharides and other components (overall concentration, 22.0 g/L).

Experiments 9 and 10 in Table 1 were performed under harsher conditions (180 and 190 °C in media containing 3% catalyst), looking for higher conversions of polysaccharides and additional delignification. In both cases, more than 95% hemicelluloses were removed from the solid phase, and %G_n_Sol fell in the range 22.7–33.3%. In particular, experiment 9 led to an OP.AD of high cellulose content (83.2%) and low lignin percentage (7.2%, corresponding to 85% delignification).

Concerning the aqueous phases, the concentrations of 1-butanol reached 76–79 g/L, near the 1-butanol solubility in water reported for the binary system [22]. HSS were the major water-soluble reaction products at 180 °C, with minor amounts of monosaccharides, furfural and 5-hydroxymethylfurfural; whereas monosaccharides were the major polysaccharide-derived products obtained at 190 °C. Despite the G_n_ and hemicellulose dissolution percentages shown in Table 2, the data in Figure 2 confirmed that the maximum amount of water-soluble products was achieved in experiment 8 (performed at a low catalyst charge). It can be noted that most furfural was transferred to the organic phase, whereas monosaccharides and HSS were almost completely kept in the aqueous phase. Although the analysis of organic phases was difficult, owing to the high dilution needed to precipitate the lignin fragments, the following conclusion could be drawn: (a) The overall monosaccharide concentrations were below 2.5 g/L in all cases; (b) most partition coefficients for acetic acid were within the range 0.8–1; and (c) the partition coefficients for furfural were around 3. Tentative material balances showed that, after stoichiometric correction, the overall amount of hemicellulose-derived products did not justify the entire hemicellulose fraction present in the feedstock, suggesting the participation of condensation reactions with other reactive species present in the media (including extractives, saccharides, furans, reaction intermediates and lignin fragments) [35,36].

On the basis of the experimental results achieved in experiments 1 to 10 in Table 1, it can be concluded that the best operational conditions corresponded to the ones of experiments 9 and 10, defined by high temperatures and catalyst charges. These results compare well with the literature reported on LCM processing in catalyzed 1-butanol–water media. Schmetz et al. [12] treated a softwood (*Cryptomeria japonica*), two hardwoods (*Eucalyptus* and beech) and tall fescue at 180 °C for 45 min in media containing 1-butanol/aqueous 1% H_2_SO_4_ (20:60 *v*/*v*). The results were strongly dependent on the feedstock: *Cryptomeria japonica* was scarcely susceptible to fractionation, retaining more than 80% of the initial lignin in the solids from treatments, but this proportion was reduced below 30% in the case of hardwoods and below 20% in the case of tall fescue. In related studies, Lancefield et al. [25] and Panovic et al. [28] considered a number of LCMs (Douglas fir wood, beech wood, walnut shells and rice husks) as substrates for fractionation in 1-butanol containing 5% water and 0.2 M HCl. Operating at the normal boiling temperature for 6 h, the solid yields ranged from 35% (in the case of walnut shells) up to 57% (in the case of Douglas fir), and the recovery of soluble lignin-derived products accounted for 17–32 g/100 g biomass. The recovered lignins showed high butoxylation levels at the β-O-4 and α-positions, but no data regarding the composition of the treated solids were provided. In a follow-up study, the same research group [32] treated rice husks under the conditions cited above and considered the scalability of the process from assays conducted with substrate amounts within the range 4–4000 g. The major products derived from hemicelluloses were both native and butoxylated monosaccharides. Del Rio et al. [26] treated beetle-killed lodgepole pine at 170 °C for 60 min in media containing 65% BuOH, H_2_SO_4_ and water, achieving solids containing 74.6% G_n_, 18.01% KL and low hemicellulose proportion; however, the selectivity of treatments could not be assessed with the available information. Zijlstra et al. [27], in a study focused on lignins, processed walnut shells, beech wood, reed grass and Douglas fir wood in a flow-through reactor, operating at 120 °C in a 9:1 1-butanol–water mixture containing 0.18 M H_2_SO_4_. The lowest lignin recovery (44%) corresponded to Douglas fir, and the maximum one (96%) was achieved using beech wood. As before, the selectivity of component separation could not be assessed because key data were missing. Amiri et al. [29] treated rice straw at 180 °C for 3 h in media containing 0.5% H_2_SO_4_ and equal volumes of 1-butanol and an aqueous solution containing 0.3 g of NaCl/1.3 g solution, leading to the formation of furfural and HMF at yields around 90 and 60 g/kg straw, respectively. In this study, the fate of lignin was not considered. Ghose et al. [30] processed rice straw in media at 120 °C for 1 or 2 h in media containing butanol and water (1:1 *v*/*v*) and 0.5% catalyst. In experiments with diverse catalysts (HCl, acetic acid, oxalic acid, aromatic compounds, anthraquinone, and a proprietary aromatic catalyst), the delignification percentages were within the range 56.2–82.8%. Using best catalyst under the same conditions, treatments of diverse LCMs (rice husks, banana stalks, wheat straw and jute sticks) led to delignification percentages of 69.3, 62.3, 79.4 and 36.2%, respectively. In this study, the fate of hemicelluloses was not considered, and the available information did not allow the evaluation of the separation selectivity. Salapa et al. [31] considered the fractionation of wheat straw at 160 or 180 °C for 20–40 min in media containing 1-butanol and acidified water at 1:1 volume ratio (H_2_SO_4_ concentration, 23 mmol/L). The treated solids contained 17.9–23.2% lignin, 67.1–71.0% cellulose and 0.4–3.3% hemicelluloses; whereas the generation of glucose and glucooligosaccharides from cellulose accounted for 9.6–19.9% of the initial cellulose, and the joint recoveries of cellulose and cellulose-derived products (glucooligosaccharides and glucose) accounted for 82.2–99.8% of the theoretical value. Teramura et al. [33] treated sorghum culms at 180 °C for 45 min in media containing 1-butanol (12.5%), water and sulfuric acid (1%), reaching 64.7% delignification at 82% cellulose recovery, as well as water soluble products derived from hemicelluloses, which were dominated by xylose, with minor amounts of glucose, furfural, HMF and acetic acid. In a further study [34], the same authors considered the effects of several operational variables (composition of media, temperature and reaction time) on the fractionation of sorghum culms. The best operational conditions (25% butanol, 0.5% sulfuric acid, 200 °C and 60 min process time) allowed the manufacture of solids (containing 84.9% cellulose, 3.6% hemicelluloses and 15.3% lignin), which kept more than 90% of the cellulose present in the feedstock.

#### 3.2.2. EF.AD Processing in Catalyzed Media Containing Water and 1-Butanol

The data reported for LCM fractionation in 1-butanol–water media included a number of substrates pre-processed by physical methods or from which juice was extracted by physico-chemical or mechanical crushing to recover the juice, including pre-soaked rice straw [30], extractive-free walnut [37], extracted vine shoots [11], brewer’s spent grains [38], sugar beet pulp [12] and sugarcane bagasse [12].

**Figure 3:**
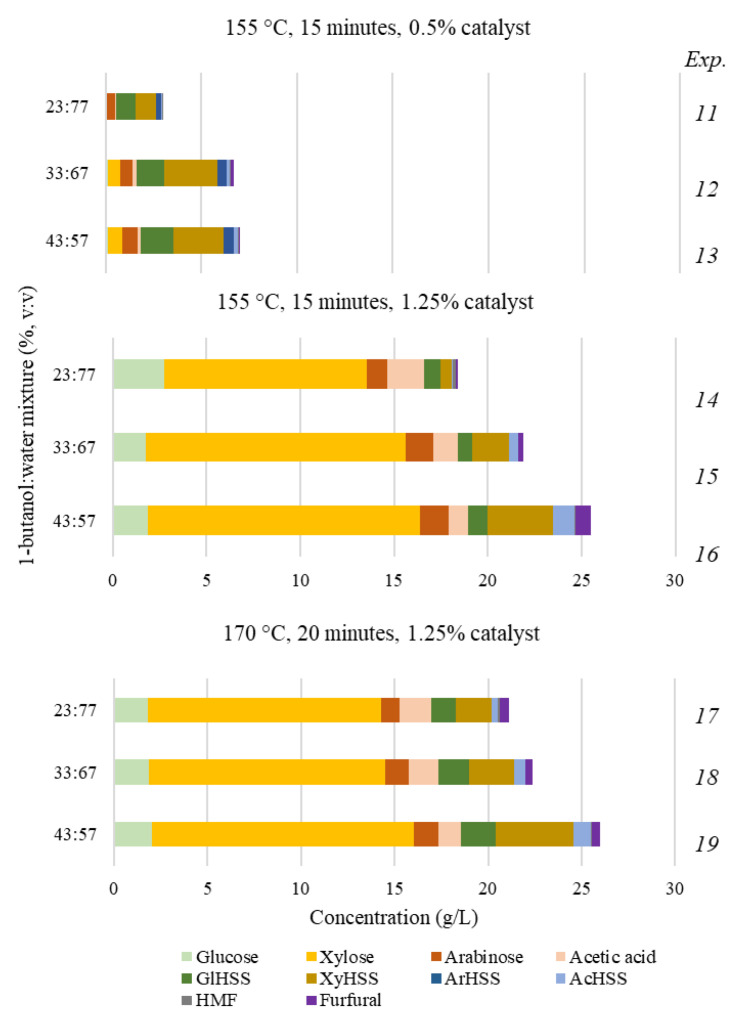
Concentrations of polysaccharide-derived products in the aqueous phases from EF.AD processing obtained in experiments 11–19 in Table 3. Nomenclature: GlHSS, anhydroglucose units in higher soluble saccharides; XyHSS, anhydroxylose units in higher soluble saccharides; ArHSS, anhydroarabinose units in higher soluble saccharides; AcHSS, acetyl groups attached to higher soluble saccharides.

The experimental plan designed to study the EF.AD fractionation is shown in Table 3. Since EF.AD is expected to be more susceptible to fractionation than AD, the first experiments (11 to 13) considered mild operational conditions (155 °C, 0.5% catalyst), and the reaction time was fixed at 15 min. Additionally, the volume percentages of 1-butanol and water were fixed at three levels (23:77, 33:67 and 43:57), looking for increased conversions of hemicellulose and/or lignin with limited cellulose losses. The results in Table 3 indicate that the conditions of these experiments were too mild for practical purposes, as it was confirmed by the moderate substrate solubilization achieved (defined by OP.FE.AD yields within the range 80.0–83.0%, with G_n_ contents below 43% and KL contents above 21%). For the sake of simplicity, the overall hemicellulose content of the solid phases was reported in terms of a single variable (TotHem). Limited effects were associated to the proportions of solvents in the reaction media, whereas the data in Table 4 confirmed a poor delignification (%LSol within the range 19.5–27.3) and a limited extent of hemicellulose dissolution (%HSol of 21.4–27.1); whereas the liquid phases contained moderate concentrations of polysaccharide-derived compounds (6.9 g/L), among which HSS were the most important components (see Figure 3).

Looking for improved results, experiments 14–16 were performed under harsher conditions, operating at the same temperature, reaction time and solvent proportions as in assays 11–13 but at an increased catalyst concentration (1.25%). The fractionation resulted more efficient, with solid yields within a practical range (41.5–42.8), higher G_n_ contents (77.2–78.7%), high degrees of hemicellulose removal (%HSol within the range 91.3–93.6%) and extensive delignification (%LSol within the range 82.0–82.7). In this subset of experiments, the highest %HSol and the lowest %LSol were achieved in the medium with the highest proportion of water, whereas the highest lignin solubilization was obtained in the 1-butanol-rich medium. Figure 3 shows that the aqueous phase from these treatments contained substantial amounts of water-soluble components, mostly corresponding to xylose. It can be noted that the concentrations of furans remain low, as both furfural and 5-hydroxymethylfurfural were mainly transferred to the organic phase.

In order to explore the effects caused for higher severities, experiments 17–19 were performed using the same catalyst charge and solvent proportions employed in experiments 14–16, but temperature and reaction time were increased up to 170 °C and 20 min, respectively. Following the general pattern described above, the water-rich medium (experiment 17) promoted the hemicellulose dissolution, leading to a solid containing 4.2% of total hemicelluloses (equivalent to 93.6 %HSol), but yielded a solid with comparatively high lignin content (10.9%, corresponding to 80.4% %LSol).

A comparative evaluation of the data in Table 3 and Table 4 confirmed that the conditions of experiment 19 (170 °C, 1.25% catalyst, 20 min) were the best ones for EF.AD processing since this assay led to a high degree of lignin dissolution (8.8% of residual KL, with 85.4 %LSol) together with an extensive hemicellulose removal (93.2% %HSol), enabling the manufacture of a processed solid (OP.EF.AD) characterized by a high G_n_ content (84.3%), while keeping a selectivity towards cellulose solubilization similar to the one determined in previous experiments. Additionally, experiment 19 led to the highest concentrations of water-soluble compounds (25.9 g/L), which were mainly composed of HSS and monosaccharides, while the furan concentrations remained low, owing to their preferential solubility in the 1-butanol rich phase.

These results compare well with the literature reported for the conversion of pre-processed LCMs in catalyzed 1-butanol–water media. Ghose et al. [30] found that presoaked rice straw was more susceptible than raw rice straw to fractionation under the conditions summarized in Section 3.2.1, leading to increased delignification (from 70 up to 80%) after 3 h of processing in the presence of a patented organic catalyst. Schmedt et al. [12], operating under the conditions indicated in Section 3.2.1, reported a higher delignification rate (87%) for sugarcane bagasse than for raw substrates treated under the same conditions, with a cellulose recovery rate above 80%. In comparison, sugarbeet pulp presented a susceptibility to fractionation intermediate between sugarcane bagasse and woods and led to a decreased cellulose recovery rate (below 70%). Migliore et al. [37] treated extractive-free walnut at 120 °C for 24 h in a medium made up of 240 mL 1-butanol, 60 mL water and 18 mL of 12 M HCl to recover α-butoxylated lignin (at 62% extraction efficiency) from the liquid phase. This study was based on lignin structure and reactivity, and the reactions undergone by cellulose and hemicelluloses were not considered. Rivas et al. [11] treated water-extracted vine shoots in media containing water, 1-butanol and sulfuric acid under a variety of experimental conditions and measured their effects on the solid recovery yields, the compositions of the processed solids and the manufacture of furans from the aqueous phases. Based on a Response Surface Methodology assessment, the authors identified operational conditions (190 °C, 20 min reaction time, media containing 52% 1-butanol and 2% sulfuric acid) for which the empirical models predicted the manufacture of solids containing 75% cellulose, with about 67% lignin removal. Foltanyi et al. [38] kept brewer’s spent grains under reflux in a mixture of 1-butanol and 4 M aqueous hydrochloric acid (95:5 *v*/*v*) for 6 h, achieving 28.5% or 32.8% recovery yields depending on the amount of substrate treated (15 or 800 g, respectively). The best solid contained 43.6% cellulose and 7.41% hemicelluloses (in comparison with 34.5% and 28.2% in the feedstock, respectively). The overall mass balance was positive (110.3–116.5 g of feedstock-derived products were recovered from 100 g of substrate), a finding ascribed to the incorporation of significant butanol amounts to the target products.

## 4. Conclusions

Both AD and water-extracted AD showed a satisfactory behavior as substrates for acid-catalyzed treatments performed in 1-butanol–water mixtures. The experimental results allowed the identification of operational conditions (corresponding to experiments 9 and 10 in Table 1 and experiment 19 in Table 2) leading to extensive delignification with intense hemicellulose solubilization and limited cellulose losses. These results compare well with literature, confirming the potential of the approach considered in this study for AD processing in the scope of lignocellulose biorefineries.

## Figures and Tables

**Table 1:** Operational conditions considered for AD fractionation and results achieved, operating at 1-butanol–water mixtures (33:67% *v*/*v*) with a liquid to solid ratio of 15 mL/g for 20 min.

Exp.	Operational Conditions		Experimental Results						
	Catalyst wt%	Temp., °C	OP.AD Yield, g/100 g AD	OP.AD Composition, wt%					
				G_n_	X_n_	Ar_n_	AcG	KL	ASL
1	1	150	75.5	40.2	21.1	0.9	3.7	20.3	2
2	1	160	69.0	45.8	20.2	0.8	3.5	18.1	1.9
3	1	170	58.8	52	15.7	0.5	2.6	17.8	1.1
4	1	180	43.9	66.6	10.3	0.2	1.4	14.5	1.4
5	1	190	36.6	77.7	4.4	0.0	0.5	10.7	1.2
6	1.5	150	73.2	41.2	20.2	0.8	3.4	19.5	1.8
7	1.5	160	61.4	49.0	16.5	0.5	3.1	18.2	1.8
8	1.5	170	47.6	62.5	10.1	0.2	1.8	15.1	1
9	3	180	33.4	83.2	3.2	0.1	0.3	7.2	1.9
10	3	190	28.7	83.7	1.6	0	0.1	8.2	1.2

**Table 2:** Values of variables defined to assess the fractionation AD under the conditions listed in Table 1.

Exp.	%G_n_Sol	%HSol	%LSol
1	15.7	22.4	17.1
2	12.1	32.5	32
3	14.9	55.9	45.2
4	18.7	79.1	65.6
5	21.0	92.8	78.6
6	16.3	28.6	23.2
7	16.5	50.9	39.7
8	17.4	76.9	62.2
9	22.7	95.2	85.0
10	33.3	97.9	86.7

**Table 3:** Operational conditions considered for EF.AD fractionation and results achieved, operating at a liquid to solid ratio of 15 mL/g.

Experiment	Operational Conditions				Experimental Results			
	Temp., °C	Catalyst wt%	Time (min)	Organic: Aqueous Phase Volume Ratio	OP.EF.AD Yield, g/100 g EF.AD	OP.EF.AD Composition, wt%		
						G_n_	TotHem	Total Lignin
11	155	0.5	15	23:77	83.0	42.0	26.1	22.7
12	155	0.5	15	33:67	80.1	42.2	25.1	21.2
13	155	0.5	15	43:57	80.0	42.9	25.3	21.4
14	155	1.25	15	23:77	41.5	78.7	4.3	10.2
15	155	1.25	15	33:67	42.8	78.6	5.4	9.8
16	155	1.25	15	43:57	42.4	77.2	5.7	9.6
17	170	1.25	20	23:77	42.3	79.4	4.2	10.9
18	170	1.25	20	33:67	40.9	83.8	4.8	9.2
19	170	1.25	20	43:57	38.8	84.3	4.8	8.8

**Table 4:** Values of variables defined to assess the fractionation of EF.AD under the conditions listed in Table 3.

Experiment	%G_n_Sol	%HSol	%LSol
11	9.9	21.4	19.5
12	12.6	27.1	27.3
13	11.4	26.6	26.8
14	15.7	93.6	82.0
15	13.1	91.6	82.1
16	15.3	91.3	82.7
17	13.2	93.6	80.4
18	11.5	92.8	83.9
19	15.4	93.2	85.4

## Data Availability

The data used to support the findings of this study are included in the article.

## References

[1] Corno L., Pilu R., Adani F. (2014). *Arundo donax* L.: A non-food crop for bioenergy and bio-compound production. Biotechnol. Adv..

[2] Angelini L.G., Ceccarini L., Bonari E. (2005). Biomass yield and energy balance of giant reed (*Arundo donax* L.) cropped in central Italy as related to different management practices. Europ. J. Agron..

[3] Licursi D., Antonetti C., Bernardini J., Cinelli P., Coltelli M.B., Lazzeri A., Martinelli M., Raspolli Galletti A.M. (2015). Characterization of the *Arundo Donax* L. solid residue from hydrothermal conversion: Comparison with technical lignins and application perspectives. Ind. Crops Prod..

[4] Mitchell R., Vogel K.P., Uden D.R. (2012). The feasibility of switchgrass for biofuel production. Biofuels.

[5] Lewandowski I., Scurlock J.M., Lindvall E., Christou M. (2003). The development and current status of perennial rhizomatous grasses as energy crops in the US and Europe. Biomass Bioenerg..

[6] Caparrós S., Garrote G., Ariza J., Díaz M.J., López F. (2007). Xylooligosaccharides production from *Arundo donax*. J. Agric. Food Chem..

[7] Caparrós S., Ariza J., López F., Díaz M.J. (2007). Optimizing cellulosic paper obtained from *Arundo donax* L. under hydrothermal treatment. J. Ind. Eng. Chem..

[8] Raspolli-Galletti A.M., Antonetti C., Ribechini E., Colombini M.P., Nassio di Nasso N., Bonari E. (2013). From giant reed to levulinic acid and gamma-valerolactone: A high yield catalytic route to valeric biofuels. Appl. Energ..

[9] Cañada-Barcala A., Rodríguez-Llorente D., López L., Navarro P., Hernández E., Águeda V., Alvarez-Torrellas S., Parajó J.C., Rivas S., Larriba M. (2021). Sustainable production of furfural in biphasic reactors using terpenoids and hydrophobic eutectic solvents. ACS Sust. Chem. Eng..

[10] Renders T., Cooreman E., Van den Bosch S., Schutyser W., Koelewijn S.-F., Vangeel T., Deneyer A., Van den Bossche G., Courtin C.M., Sels B.F. (2018). Catalytic lignocellulose biorefining in n-butanol/water: A one-pot approach toward phenolics, polyols, and cellulose. Green Chem..

[11] Rivas S., López L., Vila C., Parajó J.C. (2021). Organosolv processing of vine shoots: Fractionation and conversion of hemicellulosic sugars into platform chemicals by microwave irradiation. Biores. Technol..

[12] Schmetz Q., Teramura H., Morita K., Oshima T., Richel A., Ogino C., Kondo A. (2019). Versatility of a dilute acid/butanol pretreatment investigated on various lignocellulosic biomasses to produce lignin, monosaccharides and cellulose in distinct phases. ACS Sust. Chem. Eng..

[13] Benito P., Vaccari A., Antonetti C., Licursi D., Schiarioli N., Rodriguez-Castellón E., Raspolli-Galletti A.M. (2019). Tunable copper-hydrotalcite derived mixed oxides for sustainable ethanol condensation to n-butanol in liquid phase. J. Clean. Prod..

[14] Tobiszewski M., Namieśnik J., Pena-Pereira F. (2017). Environmental risk-based ranking of solvents using the combination of a multimedia model and multi-criteria decision analysis. Green Chem..

[15] Romo J.E., Bollar N.V., Zimmermann C.J., Wettstein S.G. (2018). Conversion of sugars and biomass to furans using heterogeneous catalysts in biphasic solvent systems. ChemCatChem.

[16] Ezeji T.C., Qureshi N., Blaschek H.P. (2007). Bioproduction of butanol from biomass: From genes to bioreactors. Curr. Opin. Biotechnol..

[17] Aguilar-Reynosa A., Romaní A., Rodríguez-Jasso R.M., Aguilar C.N., Garrote G., Ruiz H.A. (2017). Microwave heating processing as alternative of pretreatment in second-generation biorefinery: An overview. Energy Convers. Manag..

[18] Sluiter A., Ruiz R., Scarlata C., Sluiter J., Templeton D. (2008). Determination of Extractives in Biomass, NREL/TP-510-42619.

[19] Sluiter A., Hames B., Ruiz R., Scarlata C., Sluiter J., Templeton D., Crocker D. (2012). Determination of Structural Carbohydrates and Lignin in Biomass, NREL/TP-510-42618.

[20] Scordia D., Cosentino S.L., Lee J.W., Jeffries T.W. (2012). Bioconversion of giant reed (*Arundo donax* L) hemicellulose hydrolysate to ethanol by *Scheffersomyces stipitis* CBS6054. Biomass Bioenerg..

[21] Shatalov A.A., Pereira H. (2012). Xylose production from giant reed (*Arundo donax* L.): Modeling and optimization of dilute acid hydrolysis. Carbohydrate Polym..

[22] Zimmermann C.J., Bollar N.V., Wettstein S.G. (2018). Liquid phase conversion of lignocellulosic biomass using biphasic systems. Biomass Bioenerg..

[23] Jessop P.G. (2011). Searching for green solvents. Green Chem..

[24] Byrne F.P., Jin S., Paggiola G., Petchey T.H.M., Clark J.H., Farmer T.J., Hunt A.J., McElroy C.R., Sherwood J. (2016). Tools and techniques for solvent selection: Green solvent selection guides. Sustain. Chem. Process.

[25] Lancefield C.S., Panovic I., Deuss P.J., Barta K., Westwood N.J. (2017). Pre-treatment of lignocellulosic feedstocks using biorenewable alcohols: Towards complete biomass valorization. Green Chem..

[26] Del Rio L.F., Chandra R.P., Saddler J.N. (2010). The effect of varying organosolv pretreatment chemicals on the physicochemical properties and cellulolytic hydrolysis of mountain pine beetle-killed lodgepole pine. App. Biochem. Biotechnol..

[27] Zijlstra D.D., Korte J., de Vries E.P.C., Hameleers L., Wilbers E., Jurak E., Deuss P.J. (2021). Highly Efficient Semi-Continuous Extraction and In-Line Purification of High β-O-4 Butanosolv Lignin. Front. Chem..

[28] Panovic I., Miles-Barrett D.M., Lancefield C.S., Westwood N. (2019). Preparation and Reaction of β-O-4 γ-Aldehyde-Containing Butanosolv Lignins. ACS Sust. Chem. Eng..

[29] Amiri H., Karimi K., Roodpeyma S. (2010). Production of furans from rice straw by single-phase and biphasic systems. Carbohydr. Res..

[30] Ghose T.K., Pannir Selvam P.V., Ghosh P. (1983). Catalytic solvent delignification of agricultural residues: Organic catalysts. Biotechnol. Bioeng..

[31] Salapa I., Katsimpouras C., Topakas E., Sidiras D. (2017). Organosolv pretreatment of wheat straw for efficient ethanol production using various solvents. Biomass Bioenerg..

[32] Panovic I., Lancefield C.S., Phillips D., Gronnow M.J., Westwood N.J. (2019). Selective Primary Alcohol Oxidation of Lignin Streams from Butanol-Pretreated Agricultural Waste Biomass. ChemSusChem.

[33] Teramura H., Sasaki K., Oshima T., Matsuda F., Okamoto M., Shirai T., Kawaguchi H., Ogino C., Hirano K., Sazuka T. (2016). Organosolv pretreatment of sorghum bagasse using a low concentration of hydrophobic solvents such as 1-butanol or 1-pentanol. Biotechnol. Biofuels.

[34] Teramura H., Sasaki K., Oshima T., Kawaguchi H., Ogino C., Sazuka T., Kondo A. (2018). Effective usage of sorghum bagasse: Optimization of organosolv pretreatment using 25% 1-butanol and subsequent nanofiltration membrane separation. Bioresour. Technol..

[35] Huigen W.J.J., Smit A.T., de Wild P.J., den Uil H. (2012). Fractionation of wheat straw by prehydrolysis, organosolv delignification and enzymatic hydrolysis for production of sugars and lignin. Bioresour. Technol..

[36] Wang K., Yang H., Guo S., Yao X.I., Sun R.C. (2014). Comparative characterization of degraded lignin polymer from the organosolv fractionation process with various catalysts and alcohols. J. Appl. Polym. Sci..

[37] Migliore N., Zijlstra D.S., Kooten T.G.V., Deuss P.J., Raffa P. (2020). Amphiphilic Copolymers Derived from Butanosolv Lignin and Acrylamide: Synthesis, Properties in Water Solution, and Potential Applications. ACS App. Polym. Mat..

[38] Foltanyi F., Hawkins J.E., Panovic I., Bird E.J., Gloster T.M., Lancefield C.S., Westwood N.J. (2020). Analysis of the product streams obtained on butanosolv pretreatment of draff. Biomass Bioenerg..

